# PTH‐induced EndMT via miR‐29a‐5p/GSAP/Notch1 pathway contributed to valvular calcification in rats with CKD

**DOI:** 10.1111/cpr.13018

**Published:** 2021-05-04

**Authors:** Liting Wang, Rining Tang, Yuxia Zhang, Sijie Chen, Yu Guo, Xiaochen Wang, Zixiao Liu, Hong Liu, Xiaoliang Zhang, Bi‐Cheng Liu

**Affiliations:** ^1^ Institute of Nephrology School of Medicine Zhong Da Hospital Southeast University Nanjing China; ^2^ Institute of Nephrology School of Medicine NanJing LiShui People's Hospital Zhongda Hospital Lishui Branch Southeast University Nanjing China; ^3^ State Key Laboratory for Modification of Chemical Fibers and Polymer Materials International Joint Laboratory for Advanced Fiber and Low‐dimension Materials College of Materials Science and Engineering Donghua University Shanghai China

**Keywords:** chronic kidney disease, endothelial‐to‐mesenchymal transition, Notch1, parathyroid hormone, valvular calcification

## Abstract

**Background:**

Endothelial‐to‐mesenchymal transition (EndMT) is a common pathophysiology in valvular calcification (VC) among non‐chronic kidney disease (CKD) patients. However, few studies were investigated in CKD‐induced VC. Parathyroid hormone (PTH) was considered to be an important component of EndMT in CKD‐induced cardiovascular diseases. Therefore, determining whether PTH could induce valvular EndMT and elucidating corresponding mechanism involved further study.

**Methods:**

Performing a 5/6 nephrectomy with a high phosphorus diet was done to construct VC models in rats with CKD. miRNA sequencing was used to ascertain changes in microRNA in human umbilical vein endothelial cells (HUVECs) intervened by PTH. VC was observed by Von Kossa staining and scanning electron microscope.

**Results:**

PTH induced valvular EndMT in VC. Global microRNA expression profiling of HUVECs was examined in PTH versus the control in vitro, in which miR‐29a‐5p was most notably decreased and was resumed by PTHrP(7‐34) (PTH‐receptor1 inhibitor). Overexpression of miR‐29a‐5p could inhibit PTH‐induced EndMT in vitro and valvular EndMT in vivo. The dual‐luciferase assay verified that γ‐secretase‐activating protein (GASP) served as the target of miR‐29a‐5p. miR‐29a‐5p‐mimics, si‐GSAP and DAPT (γ‐secretase inhibitor) inhibited PTH‐induced γ‐secretase activation, thus blocking Notch1 pathway activation to inhibit EndMT in vitro. Moreover, Notch1 pathway activation was observed in VC. Blocking Notch1 pathway activation via AAV‐miR‐29a and DAPT inhibited valvular EndMT. In addition, blocking Notch1 pathway activation was also shown to alleviate VC.

**Conclusion:**

PTH activates valvular EndMT via miR‐29a‐5p/GSAP/Notch1 pathway, which can contribute to VC in CKD rats.

## Graphical abstract

A model depicting how miR‐29a‐5p regulating VECs EndMT. PTH could trigger a decrease in miR‐29a through PTHR1 of VECs, leading to an increase in GSAP at the protein level. GSAP further activated γ‐secretase to increase the level of NICD in the cytoplasm, which promoted the increase of HES1 and Snail expression in the nucleus to mediate the activation of Notch1 signal and endothelial‐to‐mesenchymal transition (EndMT) which cause VECs transferring to valvular interstitial cells (VICs).

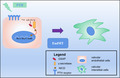

## 1 INTRODUCTION

Valvular calcification (VC), a common complication of chronic kidney disease (CKD), is more prevalent in CKD patients than in normal individuals, which mostly occurs in the aortic and mitral valves.^1^ The morbidity of VC among patients on dialysis is 28%‐50%,^2^ which severely affects the survival rate and living quality of CKD patients. Among patients undergoing long‐term dialysis, the number of calcified valves has previously been shown to be associated with all‐cause mortality and cardiovascular death. Accordingly, 1‐year all‐cause mortality was 57% with calcification of both aortic and mitral valves, 40% with either valve calcified, and only 15% for persons in whom neither was valve calcified.^1^ However, the mechanism behind VC in CKD patients remains unclear. Thus, understanding the pathogenesis of VC is of great significance in improving the prognosis of VC in patients with CKD.

Currently, the pathogenesis of VC is comprised of two aspects. One such aspect is that valvular interstitial cells (VICs) undergo cartilage/osteoid changes under stimulation of inflammation and generate osteoid/cartilage matrix.^3^ Another aspect is that valvular endothelial cells (VECs) are involved in VC by acquiring a mesenchymal or myofibroblastic (the activation type of VICs) phenotype via endothelial mesenchymal transition (EndMT).^4^ Accordingly, EndMT plays a key role in maintaining a dynamic balance between the VECs and VICs of cardiac valves. Physiologically, valvular VECs undergo EndMT and differentiate into VICs to promote the formation of valves in the embryo stage.^5^ Moreover, zebrafish mutants of Nfatc form have significantly fewer VICs due to inhibition of valvular EndMT.^6^ Among adult cardiac valves, ~1% VECs differentiate into VICs via EndMT and maintain the internal stability of the valves.^7^ However, pathologically, the balance between VECs and VICs is broken and is characterized by additional VECs undergoing EndMT in various valvular diseases with VC, such as calcified aortic valve disease (CAVD),^8^ hypoplastic left heart syndrome ^9^ and mitral valve prolapse.^10^ Inflammatory cytokine TNF‐α drives EndMT in a subset (5%‐10%) of adult VECs.^11^ Meanwhile, the results of single‐cell RNA sequencing in CAVD demonstrated that two novel valve‐derived stromal cells contributed to VC originated from VECs via EndMT, suggesting EndMT’s contribution in VC.^12^ Reportedly, blocking EndMT in VECs could slow down the process of VC; however, the contribution of EndMT to VC in a background of CKD requires further elucidation. Hence, it is necessary to explore whether VECs could undergo EndMT and aggravate VC in CKD.

Additionally, CKD, whose factors could induce and regulate valvular EndMT, also requires further consideration. Traditionally, common factors that induce EndMT of VECs include mechanical stimulation, lipid infiltration and oxidative stress.^13^, ^14^, ^15^ Compared to non‐CKD diseases, a high concentration of parathyroid hormone (PTH) was clinically reported to be associated with valve calcification in clinic. In patients who have suffered from mild to moderate CKD, the calcification degree of aortic valves was found to be related to PTH,^16^ and high PTH levels were shown to aggravate VC, eventually leading to aortic valve stenosis in CKD patients.^17^ Interestingly, in patients with primary hyperparathyroidism (PHP) without CKD, a high level of PTH exists in the body, which is directly related to VC.^18^ Such evidence demonstrates the crucial role of PTH in VC. Hence, investigating whether PTH could induce VC in CKD rats, as well as understanding its mechanism in CKD, is worth investigating. Therefore, the present study attempts to explore the role of PTH in valvular EndMT in a VC model among CKD rats.

## 2 METHODS

### 2.1 Animals

The Institutional Animal Care and Use Committee of Southeast University (Nanjing, China) authorized the present study. Eight‐week‐old male Sprague Dawley rats (Animal Laboratory of Nantong University, China) were randomly assigned to four groups: the control group (CTL) (n = 10), CKD group (n = 15), CKD+CINA group (n = 10), CKD+AAV‐29a group (n = 10), and CKD+DAPT‐treated group (n = 15). The CKD group was induced by 5/6 nephrectomy followed by a high phosphorus diet (2.0%) as per a previous study.^19^ After preforming a 5/6 nephrectomy, CKD+DAPT groups received a Notch1 signalling pathway inhibitor, DAPT, {N‐ [N‐ (3,5‐ Difluorophenacetyl) ‐l‐alanyl]‐S‐phenylglycine t‐butyl ester,} (10 mg/kg) per day for 10 weeks. The CKD+AAV‐miR‐29a group was then given an intravenous injection of AAV‐miR‐29a (2.5 × 10^11^) every three weeks, while the CKD+CIINA group was given oral CINA (10 mg/kg) daily for 10 weeks. Following the in vivo experiments, the rats were anaesthetized using an intraperitoneal injection of pentobarbital (150 mg/kg).

### 2.2 Adeno‐associated virus9 (AAV9)

A type 9 recombinant AAV‐expressing rats miR‐29a gene (AAV‐rno‐miR‐29a) was prepared by GENE. After 5/6Nx, the rats of the AAV‐miR‐29a group were given intravenous injection through the tail for every 3 weeks (2.5 × 10^11^).

### 2.3 Cells

HUVECs were purchased from ScienCell Research Laboratories (catalog number: 8000, USA) and were cultured as previously described. All experiments were performed using cells at passages 3 to 5. Cells (HUVECs) were then cultured in the presence of PTH (P3796, Sigma, 10^‐7^ mol/L) for 48 hours in order to identify the effects of PTH with/without DAPT (10 ng/mL, Sigma Aldrich) according to the phenotypic transition and differentiation capability of ECs that undergoing EndMT.

### 2.4 miRNA expression profiling

HUVECs treated with BSA (n = 3) and PTH (n = 6) were lysed, and the total RNA was extracted using Trizol reagent (TAKARA). Additionally, 250 ng of total RNA was polyA tailed and labelled with biotin with the FlashTag Biotin HSR RNA Labeling Kit (Genisphere). RNA labelling and array hybridization were performed according to Biotin's manual and were analysed using the Affymetrix 3000 GeneScanner. The significance of differentially expressed miRNAs between two groups was identified via fold change and P‐value. Hierarchical clustering was performed so as to show distinguishable miRNA expression profiling among the samples.

### 2.5 Quantitative real‐time PCR assay

Total RNA isolation was performed using Trizol by following the manufacturer's instructions (TAKARA). PCR was conducted using SYBR Premix Ex Taq and 7300 Real‐Time PCR System (Applied Biosystems), and the data were normalized to the expression of GAPDH. Primer sequences are shown in Table 1.

**TABLE 1:** Primer Sequence

	Species	Forward primer (5′‐>3′)	Reverse primer (5′‐>3′)
CD31	Rat	ATCTCCATCCTGTCGGGTAA	TGTCATTCACGGTTTCTTCG
CD10	Rat	CCTCGTTGACTGGTGGACTC	TGATAGGCTCTGTATGCTTGG
CD44	Rat	GCTCTGATTCTTGCCGTCTG	TTGAGTTCACTTGGTTTCCTGT
α‐SMA	Rat	CACTGCTGCTTCCTCTTCTTC	GATGCTGTTATAGGTGGTTTCG
FSP1	Rat	GTCCACCTTCCACAAATACTCA	GCTTCGTCTGTCCTTCTCCC
GAPDH	Rat	ACACCGACCTTCACCATCT	GGATGACTTTGGCTAGAGGAG

### 2.6 Western blot

Cells and vessel tissues were lysed in ice‐cold RIPA Lysis buffer (89901, Thermo Scientific) supplemented with protease inhibitor cocktail, and the protein concentration was determined by BCA assay (KeyGEN, BioTECH). Proteins were subjected to 10% SDS‐PAGE (Thermo Scientific) and transferred to PVDF membranes (Millipore). The membranes were then blocked with 5% w/v BSA (Biosharp) in TBST and incubated overnight with the following primary antibodies: anti‐CD31 (1:1000, ab24590, Abcam),anti‐α‐SMA (1:1000, ab32575, Abcam), anti‐FSP1 (1:1000, ab197896, Abcam), anti‐CD44 (1:1000, ab189524, Abcam), CD10 (1:1000, ab256494, Abcam), GADPH (1:3000, ab181602, Abcam), GSAP (1:1000, ab106630, Abcam), Snail (1:1000.ab216347, Abcam), NICD (1:1000, sc‐373891, Santa Cruz) and HES1 (1:1500, ab221788, Abcam). Goat anti‐mouse or anti‐rabbit secondary antibodies (Cell Signaling Technology) were used for detection on ImageQuant LAS 500(GE Healthcare). The immunoreactive bands were determined using densitometry by the Image J software (NIH).

### 2.7 Enzyme‐linked immunosorbent assay

γ‐Secretase activity was quantified via fluorescent microscopy using γ‐secretase activity kit according to the manufacturer's instructions (ImmunoTag, G‐Biosciences). Briefly, γ‐secretase activity was determined by quantification of human APH1A (Gamma‐Secretase subunit APH‐1A) with biotin‐conjugated anti‐APH1A antibody as a detection antibody. Cleavage‐dependent release was measured at 450 nm using a fluorescent microplate reader (Multiskan GO Microplate Spectrophotometer, Thermo Scientific).

### 2.8 Immunofluorescence

The slides of the valve and cells plated in confocal dishes were incubated with the primary antibody, after which they were incubated with secondary antibodies. All samples were treated with DAPI dye for nuclear staining and were detected by a confocal microscope (Olympus FV1000).

### 2.9 von Kossa

Heart sample was embedded in paraffin, sectioned and stained using the von Kossa method. The sections were then deparaffinized and incubated in 5% silver nitrate solution under ultraviolet light for 60 minutes. Unreacted silver was removed by washing with 5% silver nitrate solution for 5 minutes. The tissues were then counter‐stained with eosin for 5 minutes, thoroughly rinsed with DI water (2 mL, 3 rinses) and dehydrated in absolute alcohol (2 mL, 3 times). von Kossa staining was scored visually, with a numerical rating of 1‐5 assigned based on the following criteria: 1 = negative; 2 = rare detection; 3 = sparse but consistent; 4 = uniformly present; and 5 = intense and wide‐spread staining. Each section was reviewed by more than 2 pathologists.

### 2.10 siRNA

GSAP siRNA was purchased from Geneoharma. The non‐targeting scramble‐sequence siRNA was used as a negative control. The siRNA transfection study was performed with the MACSfectin Reagent kit (130‐098‐411, Miltenyi Biotec Inc.) and its corresponding protocol.

### 2.11 Scanning electron microscopy and energy dispersive spectroscopy

Fresh and decellularized aortic valve leaflet samples were fixed in a mixture of 2.5% glutaraldehyde and 4% paraformaldehyde at pH 7.35 for 30 minutes at room temperature. Next, the valves were washed three times with distilled water, after which they were dried using a freeze‐drying vacuum system for scanning electron microscopy (SEM) (S‐4800, Hitachi, equipped with energy dispersive spectroscopy [EDS]).

### 2.12 Statistical analysis

Data were expressed as mean ± standard deviation (SD) for each group. A two‐tailed unpaired Student's *t* test was used for comparisons between two groups, and one‐way ANOVA was performed to compare data with more than two groups followed by the Bornferroni correction for multiple comparisons. All analyses were carried out using GraphPad Prism 5.0. *P* <.05 was considered to be statistically significant.

## 3 RESULTS

### 3.1 PTH‐induced EndMT participated in VC in rats with CKD

**FIGURE 1:**
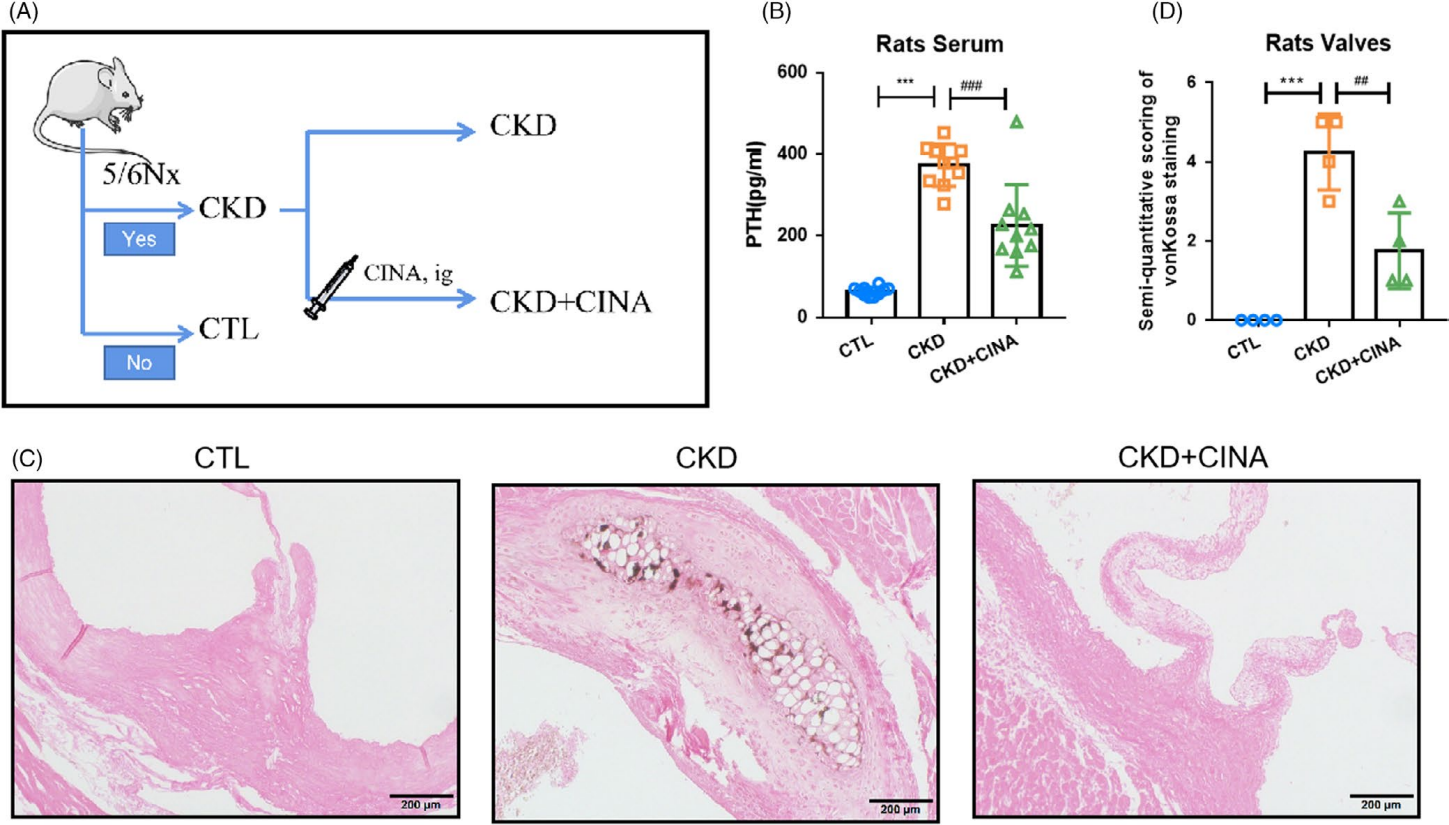
Decreasing PTH could alleviate VC in CKD rats. (A) The pattern diagram of the research design. (B) The serum levels of PTH among the CTL, CKD and CKD+CINA groups. (C, D) von Kossa staining of aortic valves of rats. ***P < .01 vs CTL group. ###*P* < .01 vs CKD group. CINA, cinacalcet; CKD, chronic kidney disease; CTL, control; PTH, parathyroid hormone

In line with our previous study,^19^ a stable VC model in rats with CKD was established (Figure 1A, Figure S1A‐C). Compared to the CTL group, serum PTH concentrations were observed to be markedly higher in the CKD group. In this study, high levels of serum PTH were attenuated by CINA (Figure 1B). As determined by von Kossa staining, the area of calcified lesions in the valves of the CKD group was found to be significantly increased compared to that of the CTL group; however, it was decreased following treatment with CINA (Figure 1C,D). The results indicated that decreasing PTH levels could inhibit VC in CKD rats.

**FIGURE 2:**
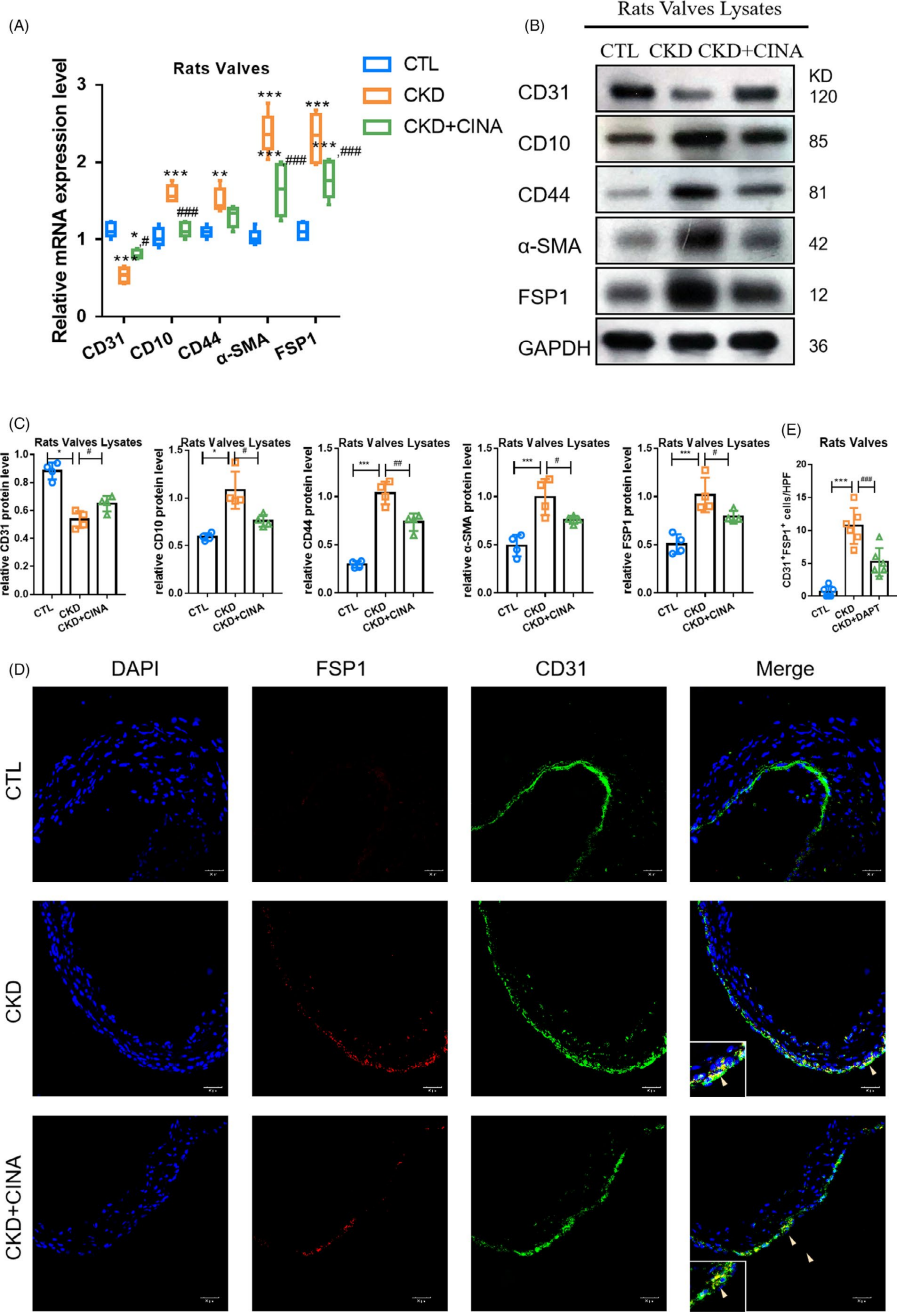
PTH‐induced EndMT in VC of CKD rats. The changes in EndMT markers in the different models. (A) mRNA levels of endothelial marker (CD31) and mesenchymal markers (α‐SMA, FSP1, CD44, CD10) in the different models. (B, C) Protein levels of endothelial marker and mesenchymal markers in different models. (D‐E) Fluorescence staining of CD31with FSP1 in aortic valves. Data were presented as the mean ± SD. n = 4 per group. **P < .01 vs CTL group. ***P < .001 vs CTL group. ^#^ *P* < .01 vs CKD group. ^##^ *P* < .01 vs CKD group

According to our previous study, PTH was considered as an important player in the influence of EndMT in CKD with cardiovascular calcification.^20^ In the present model, EndMT occurred in valves characterized by upregulated expression of mesenchymal markers (α‐SMA, FSP1, CD44, CD10) and downregulated expression of endothelial markers (CD31) at the mRNA and protein levels, which were attenuated by CINA (Figure 2A‐C). Moreover, immunofluorescence demonstrated that the number of cells both expressing CD31 and α‐SMA in the CINA group was decreased compared to that of the CKD group (Figure 2D,E). The obtained findings showed that decreasing PTH levels in serum could relieve valvular EndMT in CKD, indicating the relationship of EndMT and VC.

### 3.2 miR‐29a‐5p participated in PTH‐induced EndMT

**FIGURE 3:**
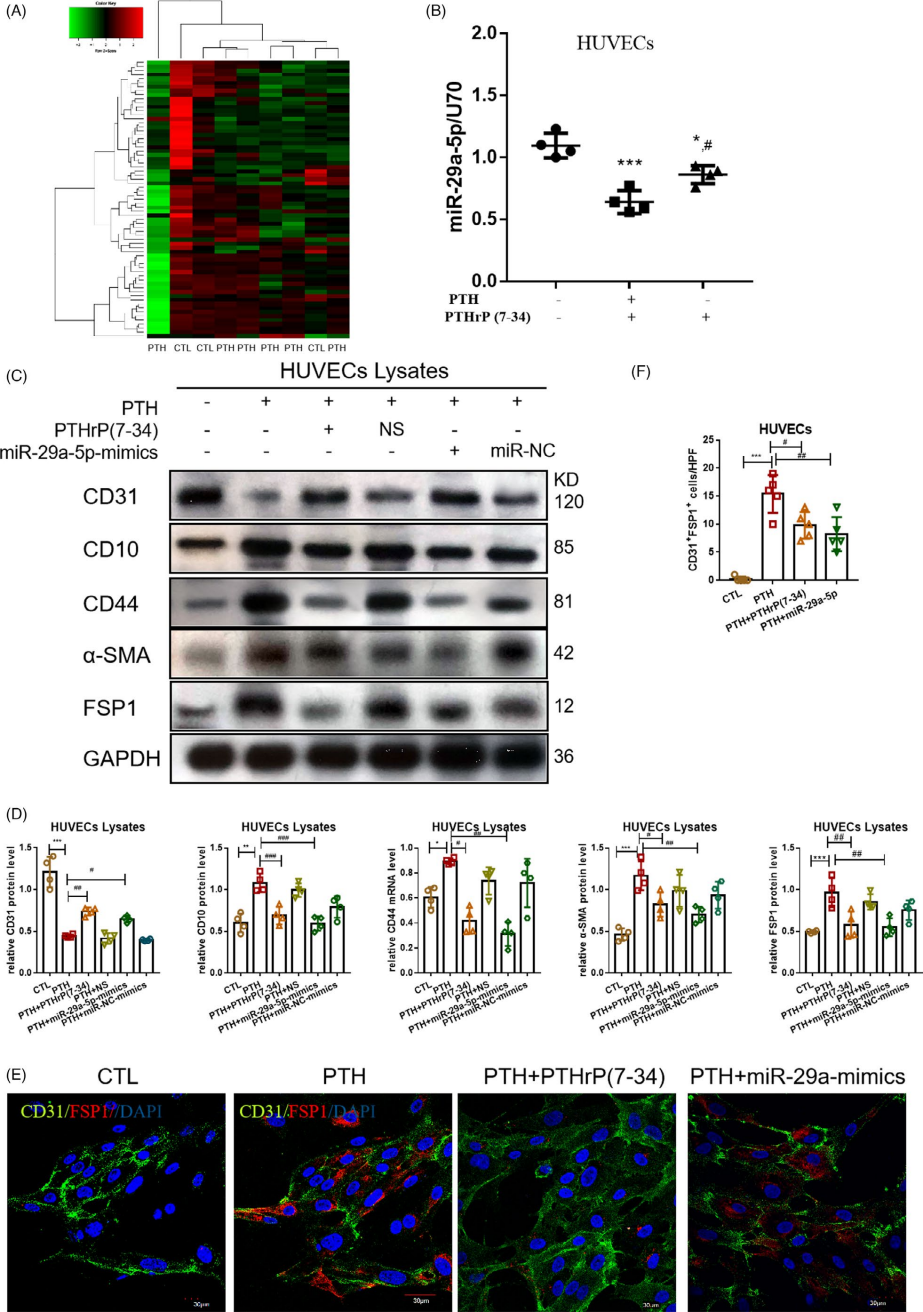
PTH‐induced HUVECs EndMT via miR‐29a‐5p. (A) miRNA sequencing in HUVECs by PTH versus CTL. CTL, n = 3; PTH, n = 6. (B) The level of miR‐29a‐5p in the groups of CTL, PTH and PTH+PTHrP(7‐34). (C, D) The protein level of EndMT markers in the CTL, PTH, PTH+PTHrP(7‐34) PTH+NS, PTH+miR‐29a‐mimics and PTH+miR‐NC groups. (E, F) Fluorescence staining of CD31 with FSP1 in HUVECs. Data were presented as the mean ± SD. n = 4 per group. *P < .05 vs CTL group. **P < .01 vs CTL group. ***P < .001 vs CTL group. #*P* < .01 vs PTH group. ^##^ *P* < .01 vs PTH group. ^###^ *P* < .001 vs PTH group

Furthermore, how PTH induced valvular EndMT was subsequently investigated. Due to the lack of commercialized human VECs, HUVECs were mainly used instead of VECs in the in vitro experiments. Accordingly, the expression profile of miRNAs in HUVECs intervened by PTH versus CTL were specifically detected, which confirmed that several HUVECs miRNAs were modulated by PTH (Figure 3A). Among them, 85 miRNAs were observed to be raised while 361 miRNAs were reduced. Additionally, levels of miR‐29a‐5p were found to be decreased compared to that of the CTL group following PTH stimulation (Figure 3B), which were resumed by PTHrP(7‐34) (PTHR1 inhibitor), suggesting that PTH‐induced miR‐29a‐5p decreased by PTHR1. Afterwards, the effects of miR‐29a‐5p in PTH‐induced HUVECs EndMT were analysed. Here, overexpressing miR‐29a‐5p or blocking PTHR1 was shown to resume the expression of endothelial markers and relieve the expression of mesenchymal markers at the protein level (Figure 3C,D). Meanwhile, in the PTH+miR‐29a‐5p‐mimics and PTH+PTHrP(7‐34) groups, the number of HUVECs both expressing CD31 and α‐SMA decreased compared to that of the PTH group (Figure 3E,F). The corresponding results suggested that the decrease of miR‐29a‐5p in HUVECs may serve as the cause of PTH‐induced EndMT.

**FIGURE 4:**
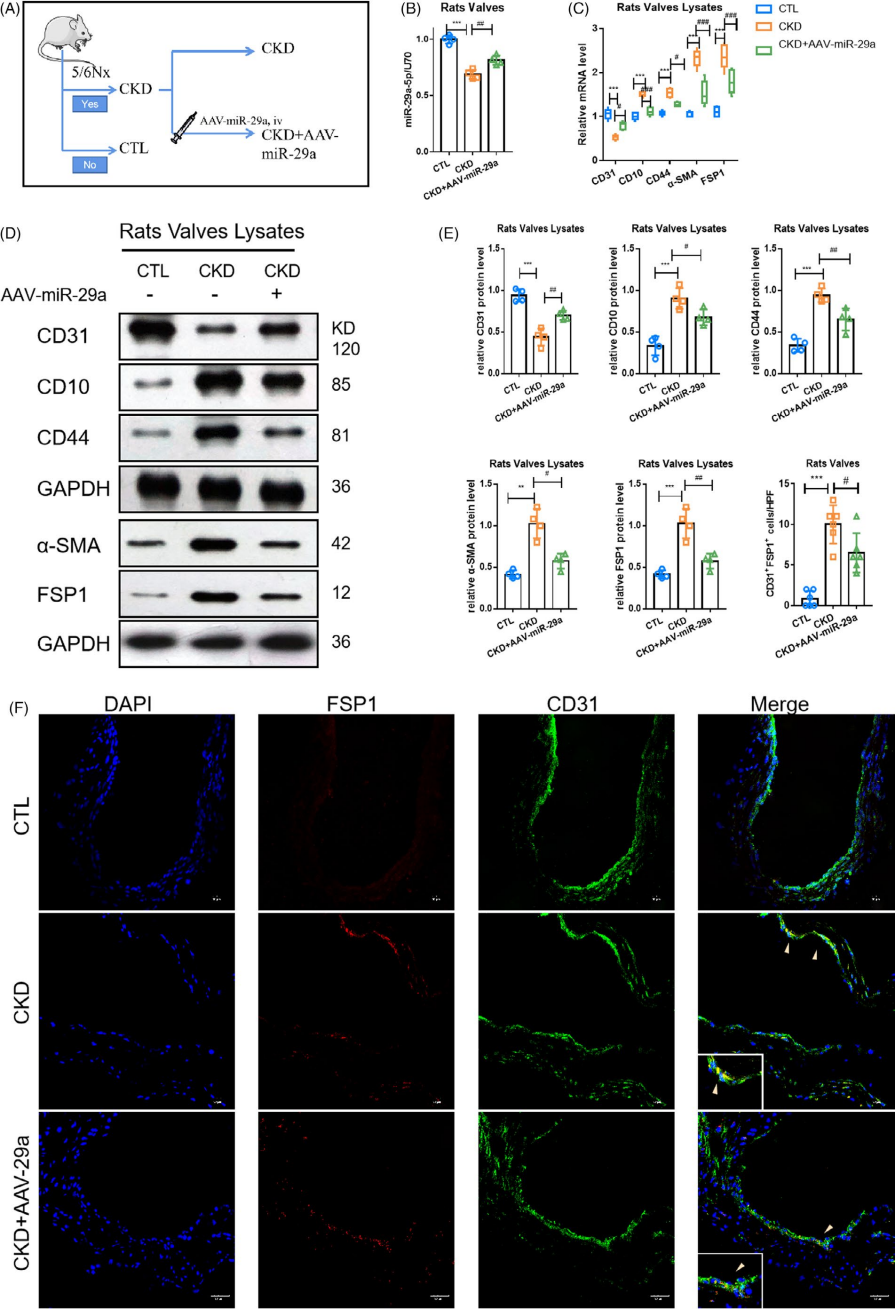
FIGUREAAV‐miR‐29a could inhibit valvular EndMT in rats with CKD. The changes in EndMT markers in different models. (A) The pattern diagram of the research design. (B) The level of miR‐29a‐5p in the CTL, CKD and CKD+AAV‐29a‐5p groups. (C) mRNA levels of endothelial marker (CD31) and mesenchymal markers (α‐SMA, FSP1, CD44, CD10) in different models. (D, E) Protein levels of endothelial marker and mesenchymal markers in different models. (F, G) Fluorescence staining of CD31with FSP1 in aortic valves. Data were presented as the mean ± SD. n = 4 per group. **P < .01 vs CTL group. ***P < .001 vs CTL group. #*P* < .01 vs CKD group. ^##^ *P* < .01 vs CKD group. ###*P* < .001 vs CKD group

In order to further study the effects of miR‐29a‐5p in vivo, a CKD rat model was also established with AAV9‐miR‐29a treatment (Figure 4A). The level of miR‐29a‐5p in the valves of CKD rats was observed to be lower compared to that of CTL, which was resumed by AAV‐miR‐29a (Figure 4B). AAV9‐miR‐29a was also shown to resume the expression of endothelial markers and relieve the expression of mesenchymal markers at the mRNA and protein levels compared to that of the CKD model (Figure 4C‐E). Meanwhile, cells both expressing CD31 and α‐SMA decreased compared to that of the CKD group (Figure 4F,G). This data suggested that miR‐29a‐5p participated in PTH‐induced valvular EndMT in CKD.

### 3.3 miR‐29a‐5p regulated GSAP/Notch1 pathway

**FIGURE 5:**
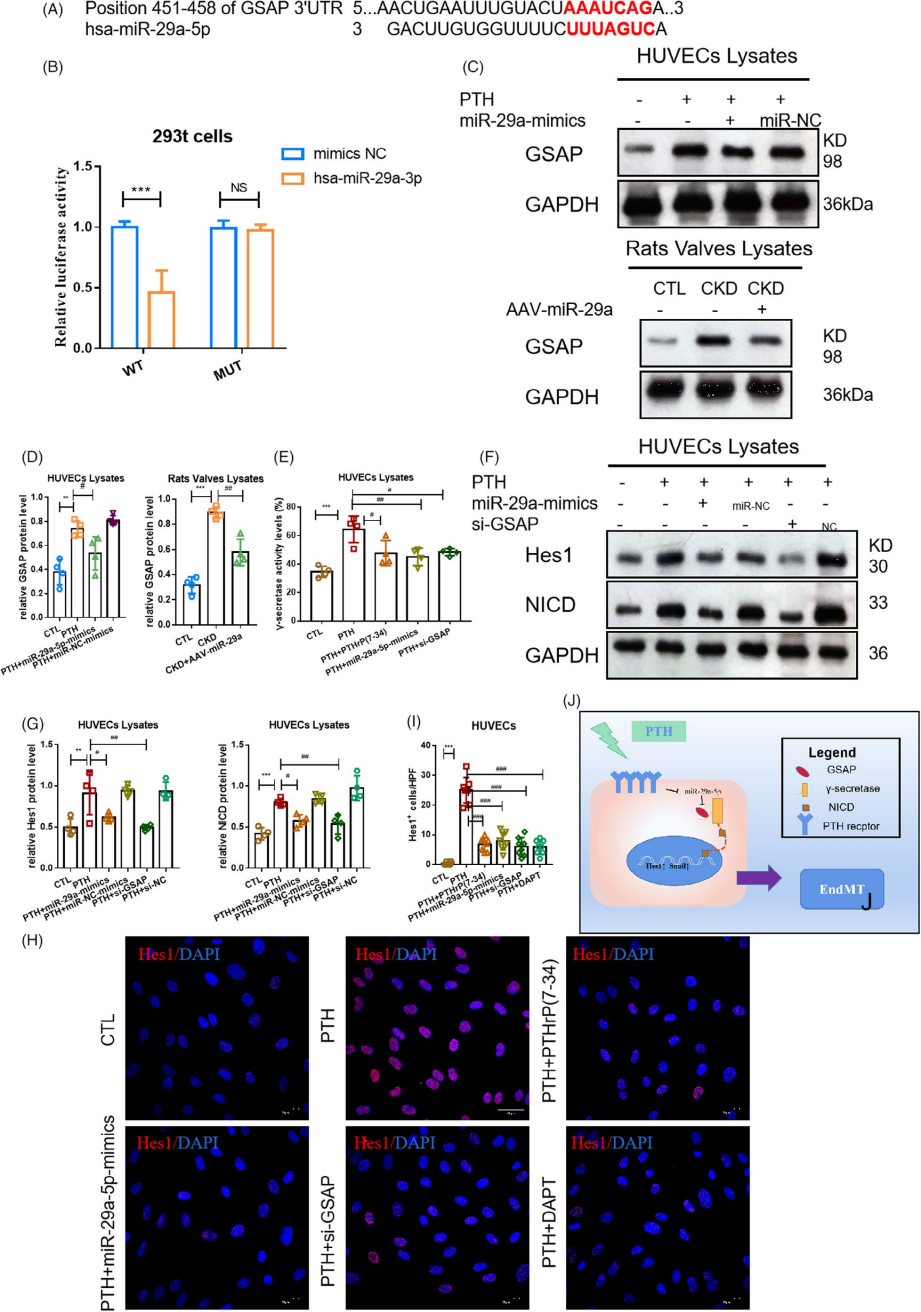
PTH‐induced Notch1 pathway activation by regulating GSAP. (A) The site prediction of miR‐29a‐5p with GSAP. (B) The dual‐luciferase assay miR‐29a‐5p with GSAP. n = 10 per group. ^***^ *P* < .01 vs mimics‐NC group. (C, D) The protein level of GSAP in groups of HUVECs and CKD rats respectively. (E) The level of γ‐secretase in the CTL, PTH, PTH+PTHrP(7‐34), PTH+miR‐29a‐mimics and PTH+si‐GSAP groups. (F, G) The protein level of NICD and Hes1 and in the CTL, PTH, PTH+miR‐29a‐5p‐mimics, PTH+miR‐NC, PTH+NS and PTH+si‐GSAP groups. (H, I) Fluorescence staining of Hes1. (J) The pattern diagram of Notch1 signal activation regulated by miR‐29a‐5p/GSAP. (C‐H) Data were presented as the mean ± SD. n = 4 per group. ^**^ *P* < .01 vs CTL group. ^***^ *P* < .001 vs CTL group. #*P* < .01 vs PTH group. ##*P* < .01 vs PTH group. ###*P* < .001 vs PTH group

The TargetScan results predicted that the γ‐secretase‐activating protein (GASP) gene may act as a potential target of miR‐29a‐5p (Figure 5A). As a result, a dual‐luciferase assay was established for verification (Figure 5B). When the miR‐29a‐5p‐mimics were co‐transfected with GSAP WT 3′‐UTR, relative luciferase activity (Renilla/firefly) was found to be significantly decreased compared to that of the group where GSAP WT 3′‐UTR was co‐transfected with miR‐NC mimics. The same effect did not occur when miR‐29a‐5p or miR‐NC was co‐transfected with GASP MUT 3′‐UTR. Moreover, the expression of GSAP was found to be upregulated upon PTH stimulation at the protein level, which was inhibited by miR‐29a‐mimics or AAV‐miR‐29a in HUVECs and CKD rats, respectively (Figure 5C,D). GSAP has been determined to modulate γ‐secretase specificity by inducing conformational change in presenilin 1 (PS1) to activate γ‐secretase, thereby inducing Notch1 signal activation.^21^ In this study, PTH was found to enhance γ‐secretase activity, which was inhibited by PTHR1 inhibitor, miR‐29a‐mimics and si‐GSAP (Figure 5E). Additionally, PTH was shown to induce Notch1 pathway activation characterized by an increased protein level of NICD (the substrate of γ‐secretase) and Hes1 (Figure 5F‐I). The corresponding data confirmed that PTH could trigger a decrease in miR‐29a through PTHR1, leading to an increase in GSAP at the protein level. GSAP was also found to further activate γ‐secretase to increase the level of NICD in the cytoplasm, which promoted the increase of HES1 expression in the nucleus to mediate the activation of Notch1 signal (Figure 5J).

### 3.4 Inhibition of miR‐29a‐5p/GSAP/Notch1 pathway could inhibit PTH‐induced EndMT in rats with CKD

**FIGURE 6:**
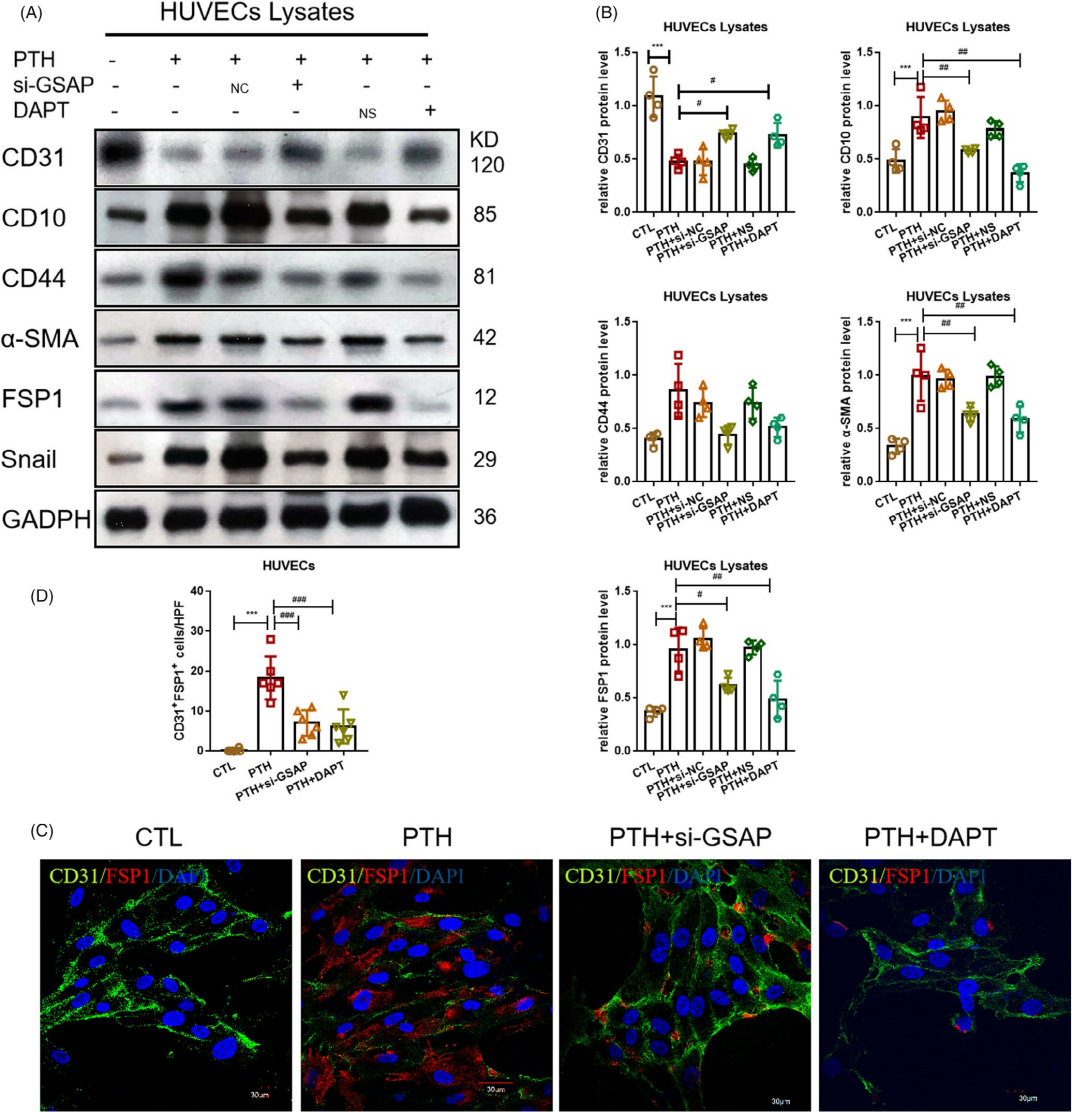
Inhibition of Notch1 pathway could inhibit PTH‐induced EndMT in vitro. (A, B) Protein levels of endothelial marker and mesenchymal markers in the CTL, PTH, PTH+si‐NC, PTH+si‐GASP and PTH+DAPT groups. (C, D) Fluorescence staining of CD31 with FSP1 in aortic valves. Data were presented as the mean ± SD. n = 4 per group. **P < .01 vs CTL group. ***P < .001 vs CTL group. #*P* < .01 vs PTH group. ##*P* < .01 vs PTH group. ###*P* < .001 vs PTH group

Next, the effects of Notch1 pathway in EndMT were investigated. DAPT [(3,5‐Difluorophenylacetyl)‐l‐alanyl‐l‐2‐phenylglycine tert‐butyl ester, C23H26F2N2O4] was applied to inhibit the secretion of γ‐secretase and indirectly inhibit the activity of NICD in vitro. miR‐29a‐5p‐mimics, si‐GSAP and DAPT were shown to resume the expression of endothelial markers and relieve the expression of mesenchymal markers (Figure 6A‐D), suggesting that inhibition of the Notch1 pathway could inhibit PTH‐induced EndMT in vitro.

**FIGURE 7:**
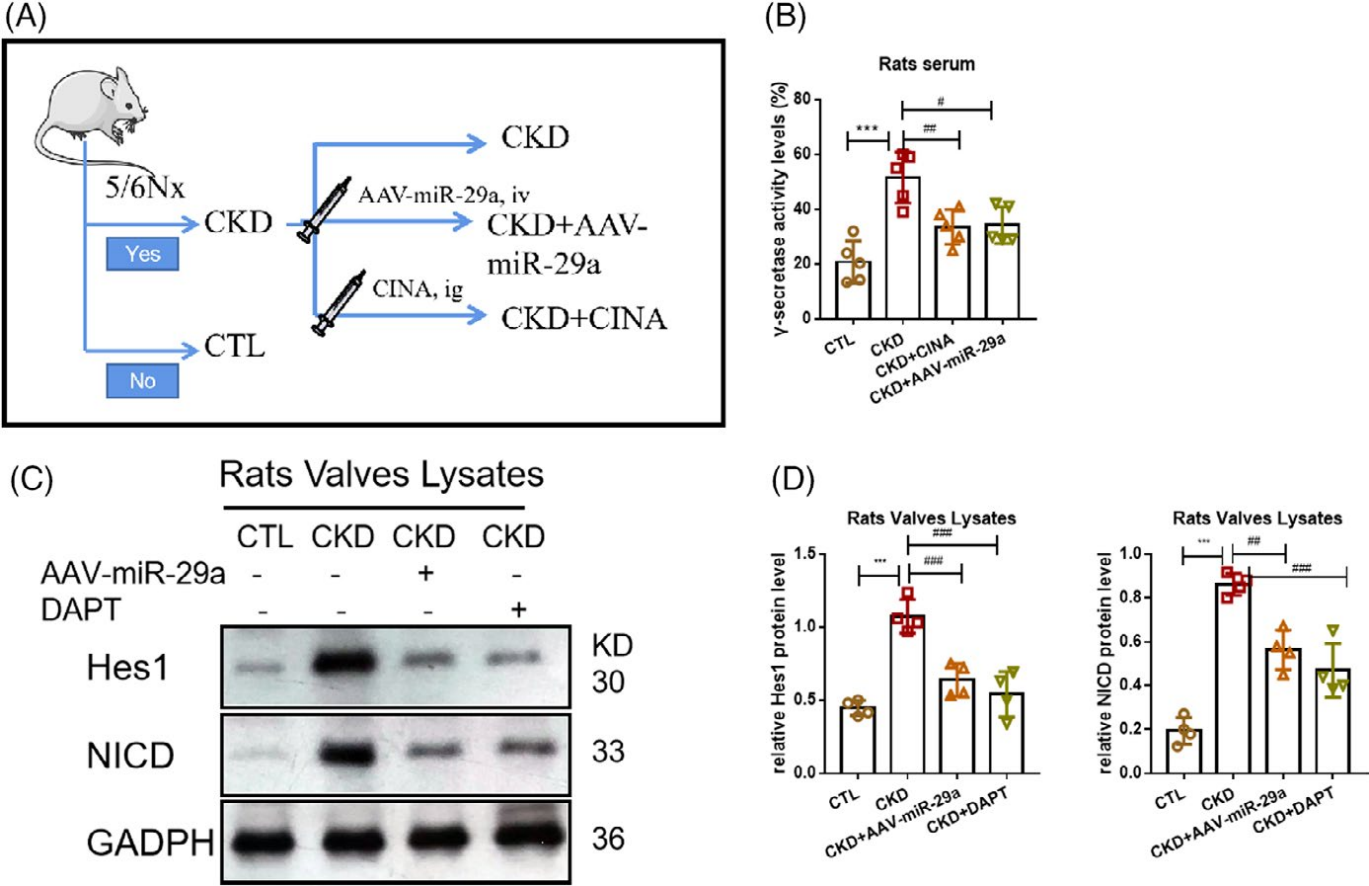
Notch1 pathway in aortic valves was activated in CKD model and inhibited by DAPT and AAV‐miR‐29a. (A) The pattern diagram of the research design. (B) The serum level of γ‐secretase activity in the CTL, CKD, CKD+AAV‐29a and CKD+DAPT groups. (C, D) The protein level of NICD and Hes1 in the CTL, CKD, CKD+AAV‐29a and CKD+DAPT groups. **P < .01 vs CTL group. ***P < .001 vs CTL group. #*P* < .01 vs PTH group. ##*P* < .01 vs CKD group. ###*P* < .001 vs CKD group

Meanwhile, DAPT and AAV‐miR‐29a were given intraperitoneal injection in order to inhibit the activation of the Notch1 pathway in vivo (Figure 7A). Compared to the CTL group, levels of γ‐secretase activity were found to be higher in the CKD group, which was attenuated by AAV‐miR‐29a and DAPT (Figure 7B). In addition, Notch1 signal activation occurred in valves characterized by the upregulated expression of NICD and Hes1, which was also relieved by AAV‐29a and DAPT (Figure 7C,D). The results indicated that AAV‐29a and DAPT could inhibit valvular Notch1 signal activation in CKD rats.

**FIGURE 8:**
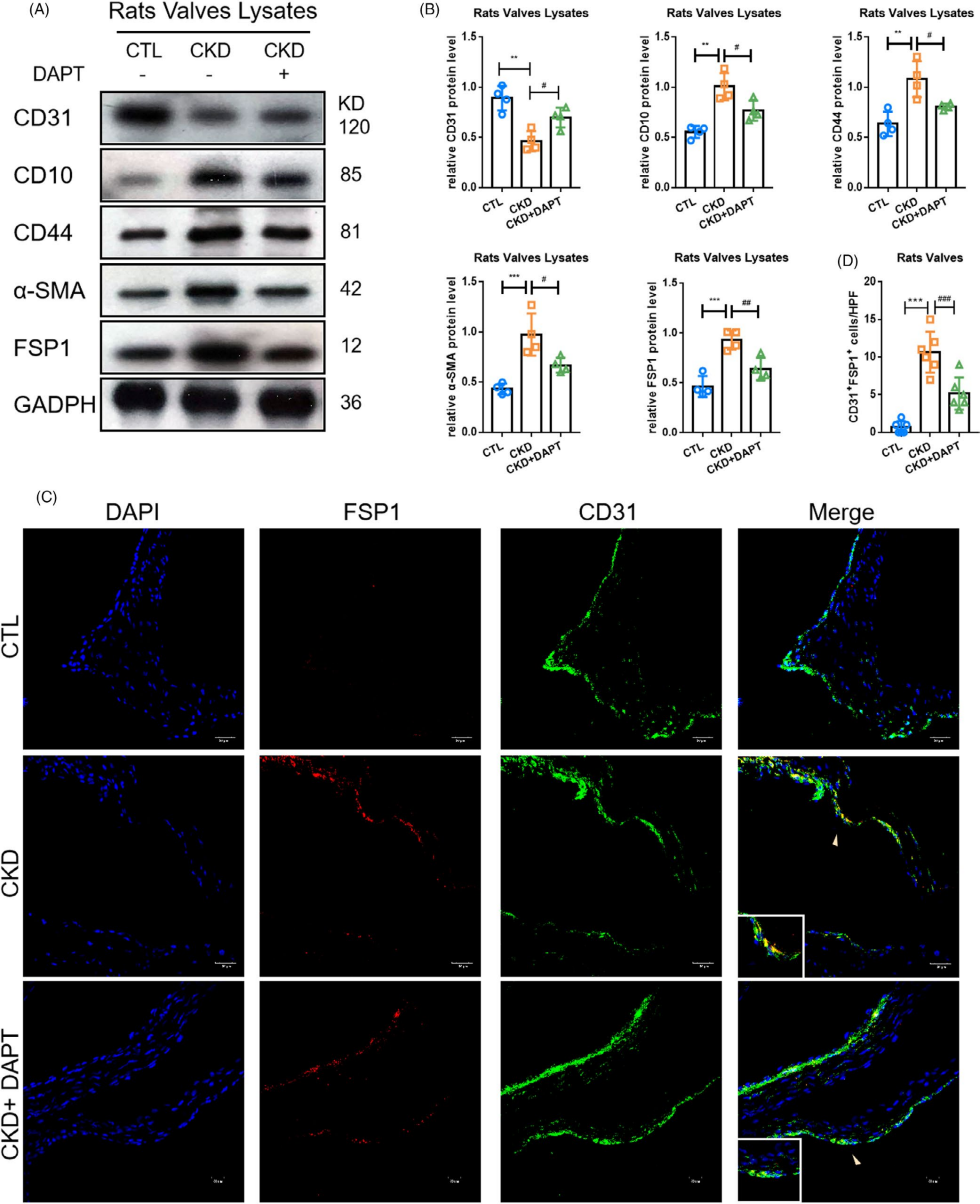
Inhibition of Notch1 activation could alleviate valvular EndMT in CKD. (A, B) Protein levels of endothelial marker and mesenchymal markers in different models. (C, D) Fluorescence staining of CD31with FSP1 in aortic valves. Data were presented as the mean ± SD. n = 4 per group. **P < .01 vs CTL group. ***P < .001 vs CTL group. #*P* < .01 vs CKD group. ^##^ *P* < .01 vs CKD group

To evaluate EndMT in vivo, the protein levels of endothelial and mesenchymal markers in valves were determined (Figure 8). The protein level of CD31 was found to be significantly lower in rat aortic valves in the CKD group compared to that of the CTL group; however, its expression was partially restored after administration of DAPT (Figure 8A,B). In contrast, mesenchymal markers (CD44, CD10, α‐SMA, FSP1) in rat valves were observed to be significantly higher in the CKD group compared to those of the CTL group, which were largely inhibited by administration of DAPT. In addition, confocal microscopy images demonstrated increased co‐localization of CD31 and FSP1 (yellow arrows) in rat aortic valves in the CKD group compared to that of the CTL group, which was eliminated by the administration of DAPT (Figure 8C,D). The corresponding findings suggest that the inhibition of the Notch1 pathway could inhibit PTH‐induced EndMT in vivo.

### 3.5 Inhibition of Notch1 pathway could delay valvular calcification

**FIGURE 9:**
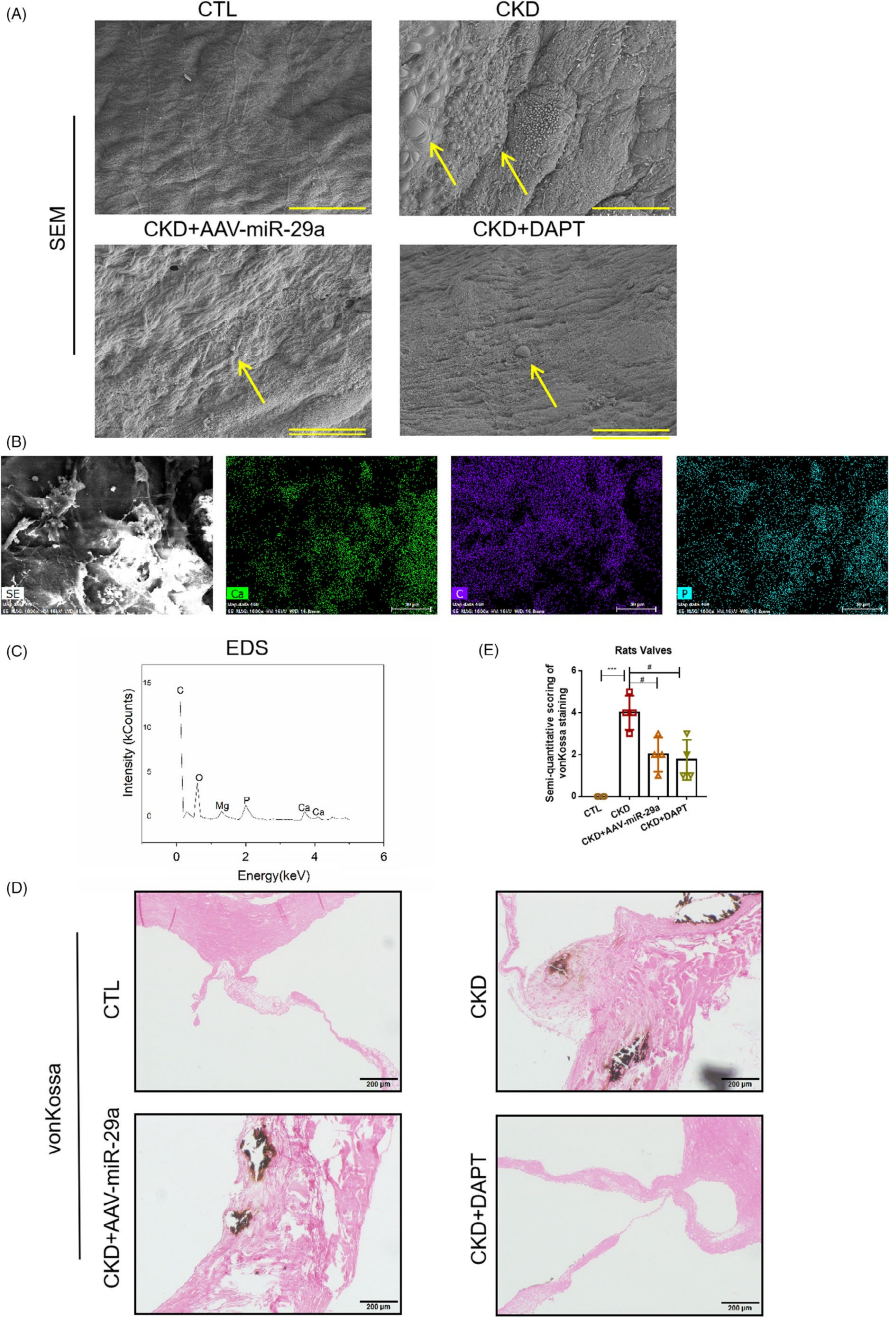
Inhibition of Notch1 pathway could alleviate VC in rats with CKD. SEM of aortic valves in different groups. (A) EDS of aortic valve in the CKD group. (B, C) The EDS of valves in CKD group. (D, E) von Kossa staining of the aortic group in different groups. Data were presented as the mean ± SD. n = 3 per group. **P < .01 vs CTL group. ***P < .001 vs CTL group. #*P* < .01 vs CKD group. ##*P* < .01 vs CKD group

EndMT has been noted to contribute to VC in various valvular diseases.^22^, ^23^, ^24^ This study attempted to investigate whether inhibiting the Notch1 pathway could delay VC in rats with CKD by blocking EndMT. Thus, we mainly focused on the effects of AAV‐29a and DAPT on VC in rats with CKD. In the micro‐structure of valves, AAV‐miR‐29a and DAPT were found to suppress the generation of the corpuscles of calcium and phosphate in valves (Figure 9A‐C). The area of aortic valvular calcified lesions was significantly enlarged in the CKD group compared to that of the CTL group, though it was reduced following AAV‐29a and DAPT treatment (Figure 9D,E). All of above data suggest that inhibition of the Notch1 pathway could delay VC in rats with CKD.

## 4 DISCUSSION

VC, a common complication of CKD, is closely correlated to cardiovascular events and death in CKD patients.^1^, ^25^ Previous studies have shown that VC is related to valvular EndMT without CKD^22^, ^23^, ^24^; however, the exact mechanisms in CKD‐induced VC remain unclear. This study mainly illustrated that: (a) high levels of PTH contributed to VC in CKD rats; (b) PTH mediated valvular EndMT by reducing miR‐29a‐5p; (c) AAV‐miR‐29a inhibited EndMT by inhibiting the activity of γ‐secretase by targeting GSAP to block the activation of the Notch1 pathway; (d) inhibition of the Notch1 pathway blocked VC in CKD rats.

EndMT of ECs is known to be an important process in cardiovascular calcification. In our previous study, PTH‐induced EndMT involved in CKD vascular calcification was found.^20^ However, the effects of PTH in VC with CKD remained unknown. One study has suggested that high PTH levels aggravated VC in the patients with mild to moderate CKD.^17^ In this paper, decreasing serum PTH level by CINA was found to inhibit VC in CKD rats, suggesting that high levels of PTH contribute to VC in CKD rats. As such, this is the first study to illustrate the role of PTH in a VC model of CKD rats. In addition, EndMT of VECs was found to participate in VC of CKD rats. Meanwhile, decreasing serum PTH levels by CINA was shown to inhibit valvular EndMT. These results indicated that PTH serves as the important driver of EndMT in VC with CKD. By conducting microRNA sequencing of HUVECs under PTH intervention, miR‐29a was shown to inhibit EndMT, and after stimulation of PTH, miR‐29a was observed to be decreased but was restored by PTHR1 inhibitor [PTHrP(7‐34)], indicating that PTH regulated miR‐29a via PTHR1. Meanwhile, in the valves of the CKD rat model, miR‐29a was found to be decreased compared to CTL, which was consistent with previous studies in non‐CKD VC diseases.^26^, ^27^, ^28^ Besides, the miR‐29a/b cluster was reported to suppress high glucose‐induced EndMT in human retinal microvascular endothelial cells.^29^ In this study, overexpression of miR‐29a was shown to inhibit PTH‐induced EndMT in the valves of CKD rats. Overall, PTH was proven to induce valvular EndMT via miR‐29a‐5p in CKD.

The potential mechanism of inhibiting EndMT through antagomiR‐29a was further investigated. In the present study, GSAP was verified to be the target gene of miR‐29a‐5p, and si‐GSAP was found to inhibit EndMT of HUVECs. Moreover, GSAP was determined to modulate γ‐secretase specificity by inducing conformational changes in PS1, thereby activating γ‐secretase to induce Notch1 activation.^21^ Activated γ‐secretase is able to independently cleave the Notch1 receptor, releasing soluble and active NICD. NICD translocates into the nucleus and binds with transcription factor RBP‐jκ, upregulates the expression of target genes (HES1,HEY1) and regulates the activity of cells.^30^, ^31^ In addition, inhibition of γ‐secretase activity by DAPT could restore TGF‐β1‐induced EndMT.^32^ Snail was thought as the main effector of EndMT in response to the Notch signal, which plays key roles in the transcriptional control of the expression of endothelial and mesenchymal markers.^33^ In this study, PTH was found to induce EndMT via miR‐29a/GSAP/Notch1 pathway in order to upregulate Snail complementing the role of miR‐29a in valve calcification with CKD.

It is widely known that EndMT participates in VC in those with non‐CKD.^22^, ^23^, ^24^ In this study, Notch1 signal was shown to participate in PTH‐induced EndMT in HUVECs. Hence, whether the inhibition of the Notch1 pathway could alleviate VC in CKD rats was further investigated. According to a previous study, abnormal Notch1 signalling activation was found to be closely correlated with osteogenic/chondrogenic signalling pathway activation as well as the calcification of mature cardiac valves.^34^ In non‐CKD VC, Notch‐activated EndMT is responsible for the initiation of aortic valve calcification.^35^ In this study, AAV‐miR‐29a and DAPT were used to inhibit Notch1 activation in order to evaluate the significance of the Notch1 pathway for VC in CKD rat models. In view of valvular morphology, AAV‐miR‐29a and DAPT could suppress the generation of corpuscles of calcium and phosphate in valves. Particles of calcium and phosphate are considered to be an important feature of the early calcification of cardiac valves.^36^ However, AAV‐miR‐29a and DAPT were found to inhibit deposition of chondroid matrix in valves, which indicated that the Notch1 pathway may serve as a key therapeutic target in VC of CKD rats. In the past, few studies have focused on CKD with VC. The study provides a new way for CKD with VC, which provides new ideas for subsequent research on targeted drugs. Nevertheless, this study has some limitations. Due to the lack of commercialized human VECs, HUVECs instead of VECs were mainly use for the in vitro experiments. Previous studies have suggested that HUVECs could serve as the primary source of artificial heart VECs.^37^ Moreover, HUVECs and human VECs undergo similar migration and proliferation as well as changes in endothelial functions in the presence of an external stimulus.^38^ Hence, HUVECs were used to replace human VECs in this study.

In conclusion, the mechanism of PTH‐induced EndMT and how it contributes to VC in CKD rats were proposed. Furthermore, this study demonstrated that PTH could induce EndMT via miR‐29a‐5p/GSAP/Notch1 pathway, and inhibition of the Notch1 signal may serve as a novel target for VC in CKD.

## CONFLICTS OF INTEREST

There are no conflicts of interest in this study.

## AUTHOR CONTRIBUTION

Ri‐ning Tang: This author is the corresponding author and takes responsibility for all aspects of the reliability and freedom from bias of the data presented and their discussion. Li‐ting Wang and Ri‐ning Tang designed research; Li‐ting Wang, Yu‐xia Zhang, Zi‐xiao Liu and Yu Guo performed research; Si‐jie Chen, Xiao‐chen Wang and Li‐ting Wang analysed data; Li‐ting Wang and Yu‐xia Zhang wrote the paper; Lin‐Li Lv, Hong Liu, Xiao‐liang Zhang and Bi‐cheng Liu reviewed and edited the manuscript.

## Supporting information

Figure S1

Legend

## Data Availability

No publicly available data or shared data are cited. All original data supporting the conclusion of the current study are available from the corresponding author on request.
