# Supplementary figures and images for: PTH‐induced EndMT via miR‐29a‐5p/GSAP/Notch1 pathway contributed to valvular calcification in rats with CKD
Source: Cell Prolif. 2021 May 4;54(6):e13018. doi: 10.1111/cpr.13018 (PMC8168417; doi:10.1111/cpr.13018)

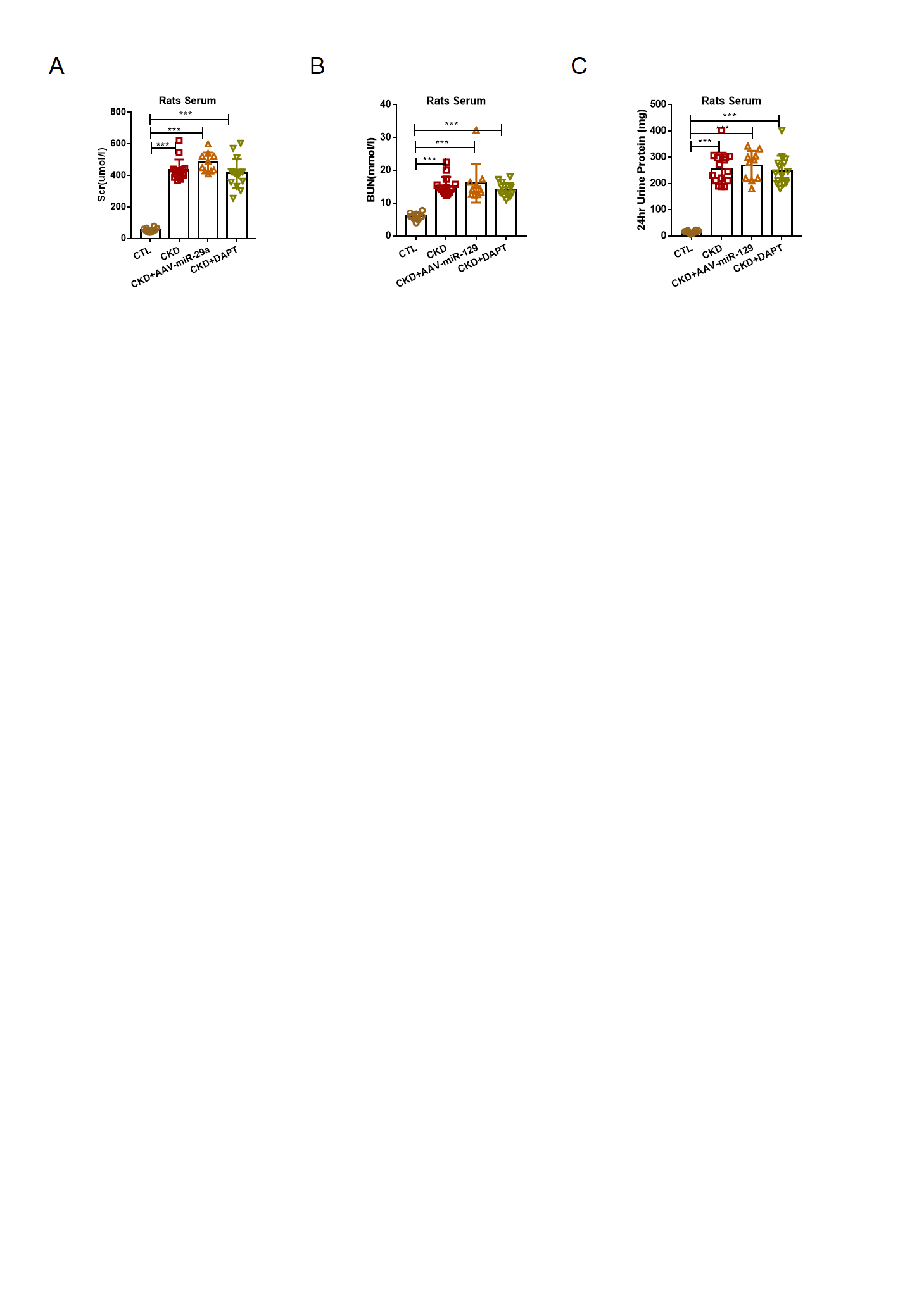

Supplement: Supplementary file 1 — Figure S1 [file CPR-54-e13018-s002.tif]
