# Supplementary material for: PTH‐induced EndMT via miR‐29a‐5p/GSAP/Notch1 pathway contributed to valvular calcification in rats with CKD
Source: Cell Prolif. 2021 May 4;54(6):e13018. doi: 10.1111/cpr.13018 (PMC8168417; doi:10.1111/cpr.13018)
Supplement: Supplementary file 2 — Legend [file CPR-54-e13018-s001.docx]

Supplemental data

Figure S1 The serological indicators of different groups. (A-C) The concentration of Scr, BUN and 24-hour protein in different groups. Scr, serum creatinine; BUN, serum urea nitrogen; 24hr Urine Protein, 24-hours urine protein. Data were presented as the mean±SD. n=3 per group.
